# 

**Published:** 1888-01

**Authors:** 


					﻿Vol. IX
JANUARY, 1888.
No. 1
TZEH-ZE
nuininuicniPiauiiinnfi-
A Monthly ’Jfour wail
DEVOTED TO
DENTAL AND ORAL SCIENCE.
W. C. BARRETT, M. D„ D. D. S.,
EDITOR.
The Hew York Dental Journal Association,
PUBDISHERS,
No. 35 West 46th Street, New York.
AND
No. 208 Franklin St., Buffalo, N.Y.
$2.50 Per Annum in Advance, .... Single Copies, 25 Cents,
Entered at the Post-Office, Buffalo, N. Y., as Second-Class matter.
				

